# Supplementary material for: Targeting the Src Pathway Enhances the Efficacy of Selective FGFR Inhibitors in Urothelial Cancers with FGFR3 Alterations
Source: Int J Mol Sci. 2020 May 1;21(9):3214. doi: 10.3390/ijms21093214 (PMC7246793; doi:10.3390/ijms21093214)
Supplement: Supplementary file 1 [file ijms-21-03214-s001.zip › Lima2020_Supplementary methods.docx]

Supplemental methods

List of primer sequences used to generate FGF3 mutations in the WT template pFB-Hyg-FGFR3.

*Primers were originally designed to generate the mutant R248A (targeted sequence AGGCC), but Sanger sequencing results showed that the generated mutant corresponded to R248G (sequence AGCGC).

| **FGFR3 mutation** | | **Primers (5' → 3')** | |
| --- | --- | --- | --- |
| **Used with QuikChange Lightning SDM Kit** | | | |
| R248C | CGC → TGC | Forward | GACGTGCTGGAG**T**GCTCCCCGCACC |
|  |  | Reverse | GGTGCGGGGAGC**A**CTCCAGCACGTC |
| S249C | TCC → TGC | Forward | ACGTGCTGGAGCGCT**G**CCCGCACC |
|  |  | Reverse | GGTGCGGG**C**AGCGCTCCAGCACGT |
| **Used with Q5 SDM kit** | | | |
| R248A* | CGC → GCC | Forward | CGTGCTGGAG**GC**CTCCCCGCAC |
|  |  | Reverse | TCCAGCGTGTACGTCTGC |
| S249A | TCC → GCC | Forward | GCTGGAGCGC**G**CCCCGCACCGGC |
|  |  | Reverse | ACGTCCAGCGTGTACGTCTGCCGGATG |
