# Supplementary figures and images for: Targeting the Src Pathway Enhances the Efficacy of Selective FGFR Inhibitors in Urothelial Cancers with FGFR3 Alterations
Source: Int J Mol Sci. 2020 May 1;21(9):3214. doi: 10.3390/ijms21093214 (PMC7246793; doi:10.3390/ijms21093214)

Supplemental Figure 1

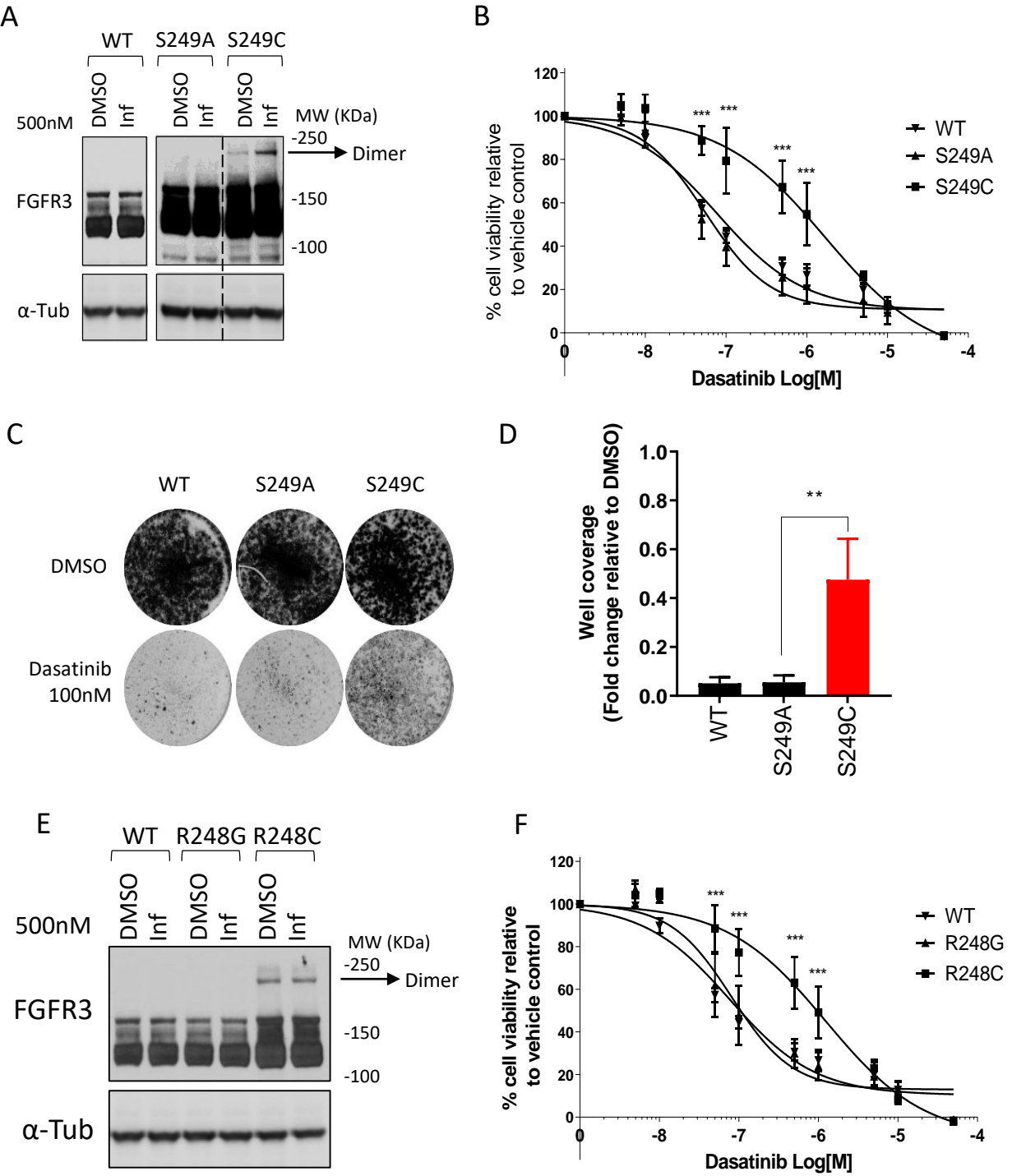

Supplemental Figure 2

A

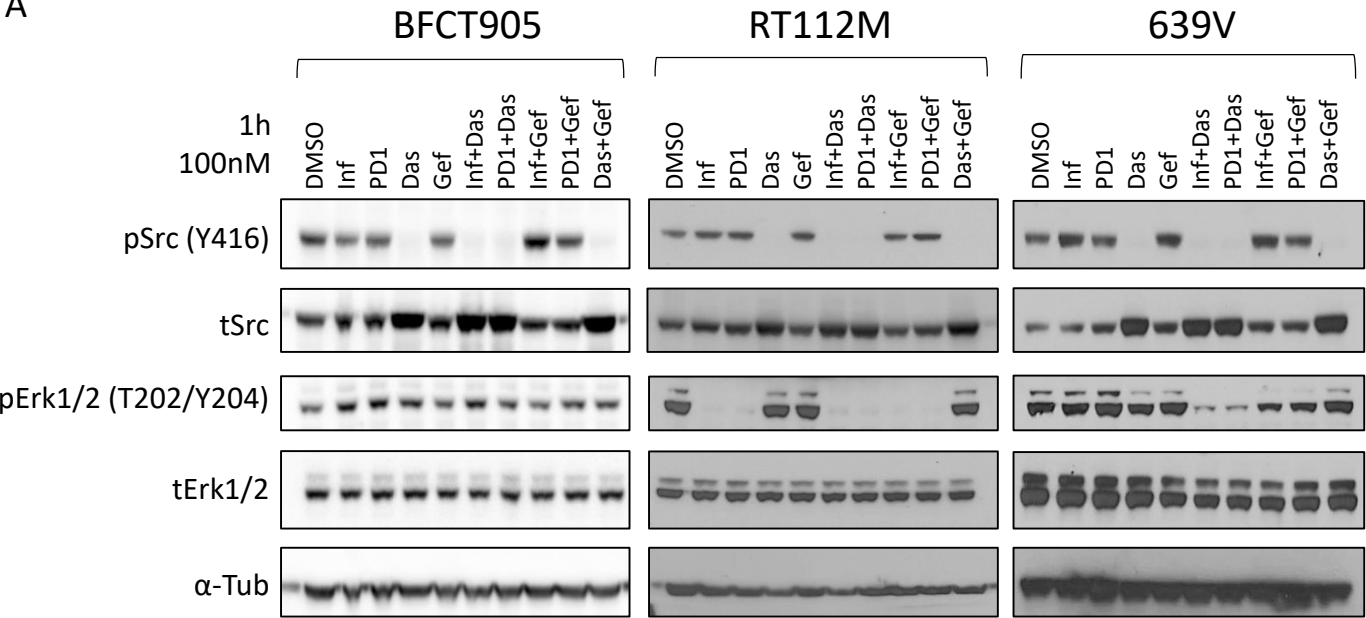

B

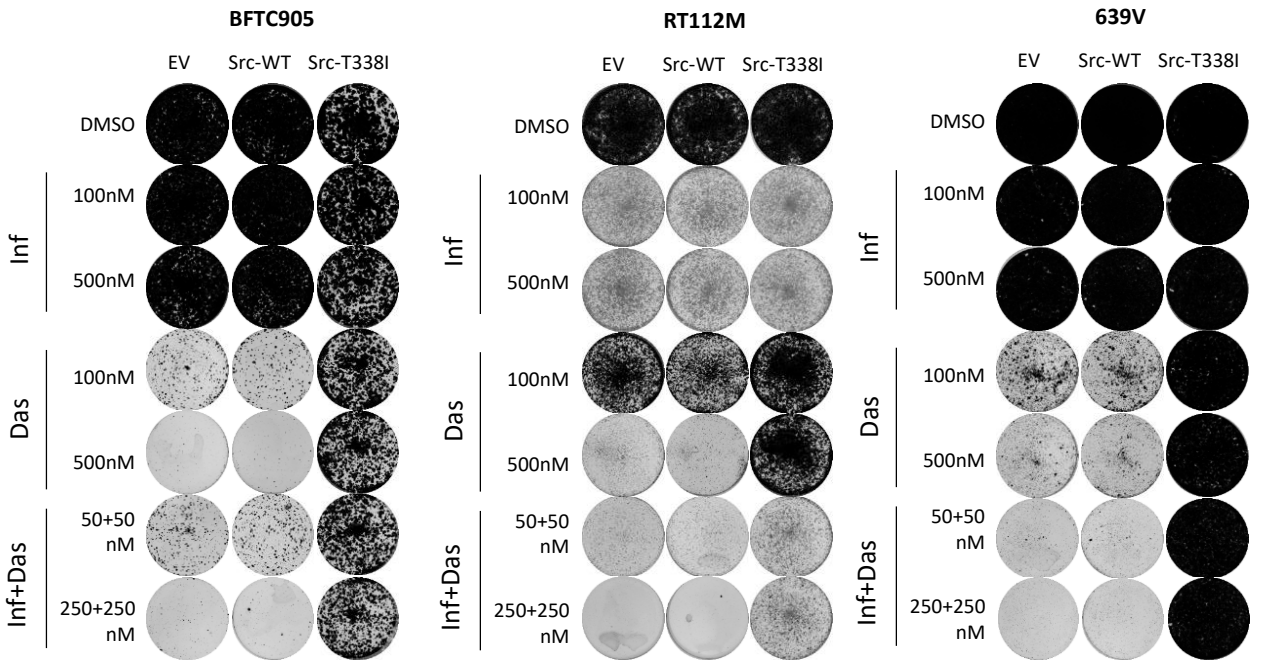

Supplement: Supplementary file 1 [file ijms-21-03214-s001.zip › Lima2020_suppfigures_final.pdf]
